# ShimNet: A Neural Network for Postacquisition Improvement of NMR Spectra Distorted by Magnetic-Field Inhomogeneity

**DOI:** 10.1021/acs.jpcb.5c02632

**Published:** 2025-06-04

**Authors:** Sylwia Jopa, Marek Bukowicki, Alexandra Shchukina, Krzysztof Kazimierczuk

**Affiliations:** † Centre of New Technologies, 49605 University of Warsaw, Banacha 2C, 02-097 Warsaw, Poland; ‡ Faculty of Physics, 427050 University of Warsaw, Pasteura 5, 02-093 Warsaw, Poland; § Faculty of Chemistry, University of Warsaw, Pasteura 1, 02-093 Warsaw, Poland

## Abstract

Despite commonly applied corrections, known as shimming, the magnetic field in an NMR spectrometer is never perfectly homogeneous. This undesired effect distorts the lineshapes, degrades the resolution, and lowers the signal-to-noise ratio in the collected spectra. As a remedy, numerical techniques have been developed to correct the spectra after acquisition, with reference deconvolution being the most popular example. However, these methods require a precarious parameter guess from the user, making them inconvenient and prone to errors. In particular, the imperfections of the reference deconvolution manifest themselves as strong oscillations at the spectral baseline. We propose a postacquisition shimming tool named ShimNet based on a convolutional neural network with the attention mechanism. The model learns the distortion characteristics of a given spectrometer from a series of calibration measurements. Once trained, it can correct any spectrum from the same machine in a fully automatic way. It achieves slightly better reconstruction quality than the existing methods and is considerably faster. This makes ShimNet an excellent tool for laboratories performing routine and massive NMR measurements for chemists. As an exemplary application, we demonstrate that the model properly corrects liquid-state spectra of small molecules, such as azarone, styrene, Cresol Red, or sodium butyrate. The open-source Python code is freely available from https://github.com/center4ml/shimnet and as the web service at https://huggingface.co/spaces/NMR-CeNT-UW/ShimNet.

## Graphical abstract

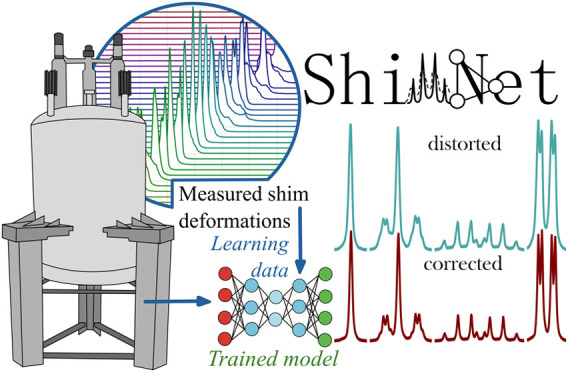

## 1 Introduction

Nuclear magnetic resonance (NMR) spectroscopy is an essential source of the present knowledge about molecular structures. It exploits the Zeeman interaction between the nuclear magnetic moments and an external magnetic field produced by the superconducting magnet of an NMR spectrometer. Modern high-resolution spectroscopy requires a very homogeneous field that must be precisely adjusted using a set of correction coils.^1,2^ They are called shims and generate individual fields, theoretically described by spherical harmonics.^3^ In practice, however, these fields are far from the ideal profiles and significantly differ between spectrometers.^4^

Apart from basic manual shimming, several automatic methods have been developed. Gradient shimming is the most popular,^5–8^ but AI-based solutions are also becoming increasingly important.^9^ Unfortunately, even highly automated procedures are still time-consuming. This is particularly annoying when hundreds of ^1^H NMR spectra are collected daily using an automatic sample changer or in other massive measurements routinely performed in industry and academia.^10,11^ Usually, only an approximate correction is applied using the same shim map for all examined samples, even if they differ in volume, alignment, or magnetic susceptibility (e.g., due to different solvents). All in all, spectra are often acquired in the presence of residual inhomogeneities, which distort the lineshapes, degrade the resolution, and lower the signal-to-noise ratio.

As a remedy, numerical techniques have been developed to correct the mis-shimmed spectra after their acquisition. The reference deconvolution (RD) is the most popular and has become the de facto standard in NMR spectroscopy.^12–15^ In this approach, the user has to select a single reference peak in each distorted spectrum and provide its proper, undistorted shape. This amounts to guessing the peak width, chemical shift, height, and the proportion of Gaussian to Lorentzian components. Then, a function reflecting the distortion is determined from the distorted reference peak via deconvolution. Finally, this function is deconvolved from the entire distorted spectrum to yield the corrected or shimmed one. The reference peak should have a relatively simple shape and be well resolved from other spectral lines. In the original approach, the peak had to be a singlet, but the method was later generalized to allow for reference multiplets as well.^16^

The main advantage of RD is its simplicity, but selecting an appropriate reference peak in each spectrum and correctly guessing its parameters may be tricky. Commercial NMR software (like Mnova 15.0.1 used in this study) can assist in determining the perfect line shape, but even such automatic predictions are sometimes inaccurate. If the reference is poorly defined, noisy, or overlaps with other peaks, RD becomes significantly less effective. Then, the correction is not perfect, and even additional distortions, such as baseline artifacts, may appear. These issues are particularly problematic for samples with low concentrations (low signal-to-noise ratio).

Recently, Xiao et al. introduced a postacquisition shimming method based on deep learning and referred to as Restore High-resolution Unet (RH-Unet).^17^ The correction is not made by a simple deconvolution but using a neural network, which gives more accurate results. Notably, many peaks can be used as a reference, improving the correction quality. Still, the method shares the main limitation of RD in that the user has to guess the undistorted parameters of the reference peaks having only the distorted spectrum at hand. Moreover, the network has to be trained for each distorted spectrum separately.

RH-UNet is an example of rapidly developing and diverse applications of deep learning in NMR signal processing. They are summarized in a recent review^18^ and include sparse sampling reconstruction,^19–23^ pure-shift spectroscopy,^24–26^ peak-picking,^27–31^ denoising,^32–34^ etc. The present paper introduces ShimNet, a tool that also fits into this trend.

ShimNet is the first postacquisition shimming method that does not require the spectroscopist to select any reference peak or guess its parameters. The solution is based on a convolutional neural network with an autoencoder architecture and uses the attention mechanism. The network learns from a series of calibration measurements how spectral lines can be distorted by magnetic-field inhomogeneities in a given spectrometer. Then it employs the acquired knowledge to correct other spectra from the same machine. The calibration experiment and the training process do require user involvement but once the network is trained, it applies the correction in a fully automatic way, without any effort from the spectroscopist.

In a typical NMR laboratory, technical staff can conduct the time-consuming calibration experiments and train the ShimNet neural network. The end user obtains the trained model and only runs it to correct the collected spectra. In particular, the correction is very fast and can easily be performed for massive amounts of measurements. The method also achieves slightly better reconstruction quality than RD and RH-Unet. The Python code is open-source and freely available from github and as the web service at https://huggingface.co/spaces/NMR-CeNT-UW/ShimNet.

In Section 2, we describe the method in detail and in Section 3, we give a brief overview of the ShimNet software package. Section 4 demonstrates that ShimNet properly corrects spectra of small molecules, such as azarone, styrene, sodium butyrate, or Cresol Red, in liquid solutions. Finally, Section 5 summarizes the main conclusions and gives possible directions for further improvements.

## 2 Methodology

We start presenting our approach with Section 2.1, which gives experimental details of our calibration and test measurements. We then come to the ShimNet model itself. Section 2.2 explains how to use the network once it is trained, Section 2.3 details the network architecture, and Section 2.4 describes all the steps necessary to train the model.

### 2.1 Experimental Details

In the present work, we use an Agilent 700 MHz and an Agilent 600 MHz spectrometers, both with DirectDrive 2 consoles. All discussed spectra were collected in a single scan using a 90° pulse at 25 °C. The spectral width was set to 11160.71 Hz (700 MHz machine) or 9615.4 Hz (600 MHz machine) and the number of complex time-domain points to 32,768, yielding a frequency resolution of 0.34 Hz (700 MHz machine) or 0.29 Hz (600 MHz machine). These acquisition parameters were identical in the calibration measurements described in Section 2.4.1 and in the test experiments of Section 4.

We perform the calibration measurements of Section 2.4.1 for 1% chloroform solution in deuterated acetone, provided by the spectrometer vendor as the standard shimming sample. The ^1^H signal from CHCl_3_ has an extremely high transverse relaxation time of ca. 20 s which brings practically no contribution to the experimental line width even on a well-shimmed spectrometer. This is appropriate for our use case because the calibration peaks should be as narrow as possible.

Due to the large number of calibration spectra, we used the nmrglue library^35^ to fully automatize the preprocessing routine in a Python script. The preprocessing was fairly standard and consisted of Fourier transform followed by automatic phase correction (0th order). Zero-filling was not applied, so that the original frequency resolution was not altered in preprocessing.

Samples for the test experiments of Section 4 were prepared in 5 mm economy NMR tubes from Deutero GmbH. We used Mnova software (Mnova 15.0.1, Mestrelab Research, S. L., Spain) to perform preprocessing that included Fourier transform, automatic phase correction (0th and first order), and baseline correction with Bernstein polynomials (third order).

Our experimental conditions are representative for a broad class of measurements routinely performed in industry and academia. We intentionally employ slightly different preprocessing methods for the calibration and test experiments to demonstrate that the ShimNet model is universal and not sensitive to such details.

### 2.2 Postacquisition Shimming with ShimNet

Once ShimNet is trained, its use is very simple. First, the raw spectrum must be preprocessed in a standard way that may include zero-filling and baseline correction, if necessary. The proper phasing is essential. Apodization disturbing the Gaussian–Lorentzian lineshapes should be avoided. Any software can be used for preprocessing as long as the results are reasonable. The preprocessed spectrum is distorted due to imperfect shimming. Technically, it constitutes a vector of real numbers. This vector is directly fed to ShimNet as input, and the network outputs another real vector of the same size, containing the corrected spectrum. If necessary, the corrected spectrum can be processed with Hilbert and inverse Fourier transformations to allow further time-domain processing. Figure 1 shows an example spectrum region before and after correction.

**Figure 1:**
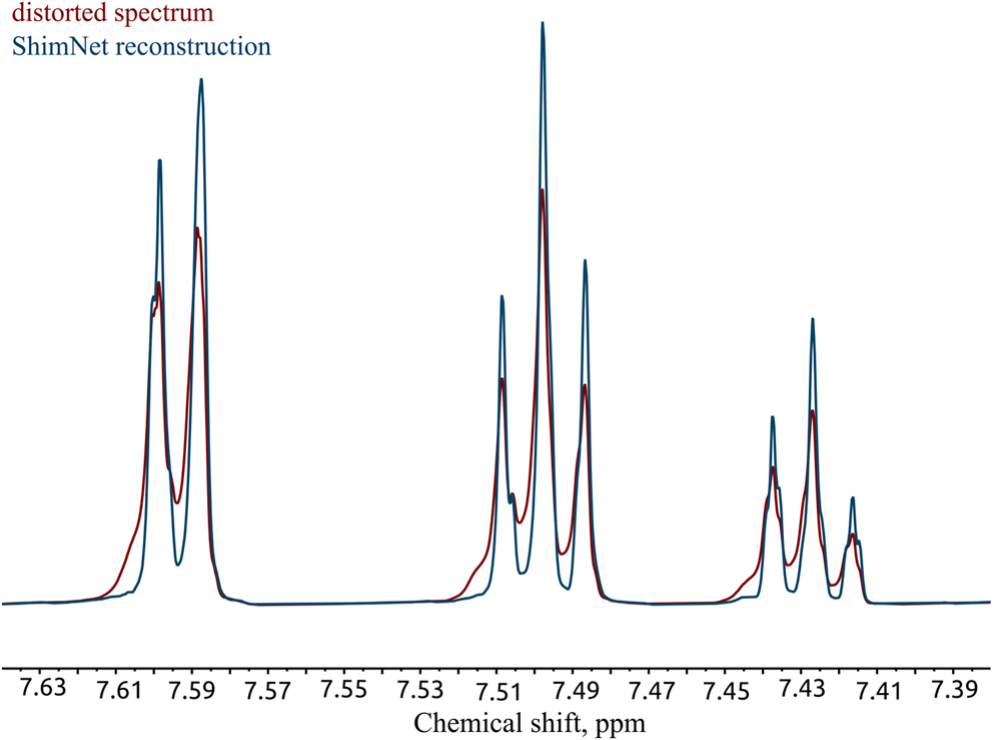
Reconstruction (blue) of a sample distorted spectrum (red) with ShimNet. The figure shows a region in a ^1^H NMR spectrum of 3 M styrene in CDCl_3_ acquired on a 700 MHz spectrometer.

The program automatically rescales the preprocessed spectra to match the peak-height range for which the neural network is prepared in the training phase. It can process spectra of any length but the frequency resolution must match that used in the training phase, as explained in Section 2.4.3.

### 2.3 Neural Network Architecture

Our neural network consists of four parts depicted in Figure 2: The convolutional encoder, the attention layer, the convolutional decoder, and an auxiliary fully connected decoder. The distorted spectrum is fed to the encoder, and the main decoder outputs the corrected one.

**Figure 2:**
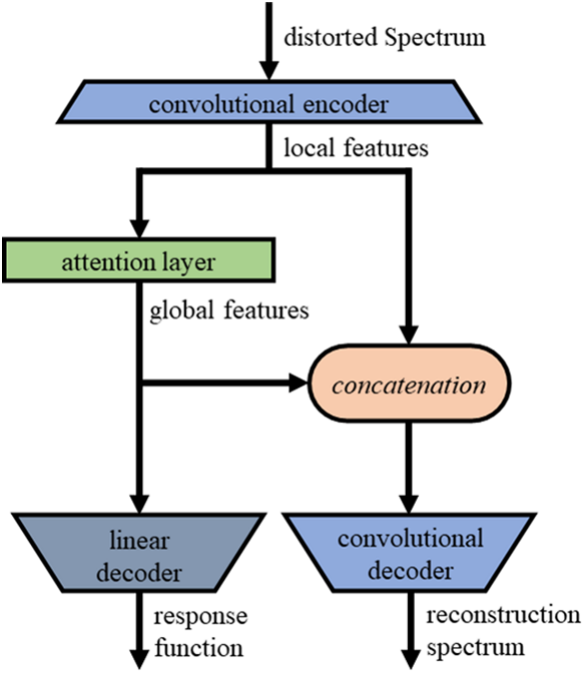
Architecture of the ShimNet neural network.

The encoder contains four one-dimensional convolution layers, each with a kernel of 7 points and 64 channels, interleaved with three ReLU activations. It has a receptive field of 25 points so that every 25 consecutive points of the input spectrum are independently encoded as a vector of 64 latent features. The main decoder is a mirror reflection of the encoder and uses transposed convolutions instead of traditional ones. The decoder input is attached to the encoder output to form an architecture known as an autoencoder. It reconstructs each point based on its 49 adjacent and symmetrically distributed neighbors. This solution is designed to capture the local behavior of the input spectrum.

Like in the case of RD and RH-Unet, our approach is based on the assumption that the entire spectrum is distorted in the same way. However, it is often better to extract information about such global distortion from other, distant parts of the spectrum, where cleaner peaks are located. To achieve this, we resort to the attention mechanism, initially considered in the context of machine translation and recurrent networks,^36,37^ then successfully applied to multiple-instance learning^38^ and finally used as a foundation of the renowned transformer architecture.^39^ Our implementation is the most straightforward linear attention of Ilse et al.,^38^ which calculates scalar products with a single query vector.

The attention layer takes the 64 latent features from each 25-point receptive field in the input spectrum and assigns a weight to each of these fields. Receptive fields that carry more valuable information for the reconstruction task are expected to get higher weights. Then, these weights are used to calculate a weighted average of latent features from all the receptive fields in the spectrum. The resulting average is a single vector of 64 features that characterize the entire spectrum and contain information about the global distortion.

This common vector is then concatenated with an individual vector of latent features from every receptive field. Thus, each field now has 128 features containing both local and global information. They are passed to the decoder, which gets twice more input channels but otherwise remains intact. The attention layer has 64 free parameters determined in the training process together with all the other parameters of the network. The network has 202,176 parameters in total.

The attention mechanism presumably prioritizes receptive fields that contain clear information about distortion. Broad peaks, whether singlets or multiplets, may be less affected because the natural line width masks the distortion. In contrast, narrow peaks—especially singlets—exhibit more distinct and recognizable deformations, making them more suitable for extracting information about distortion. Multiplets, although potentially informative, can be challenging to analyze because of their complexity. Thus, narrow singlets consistently receive the highest attention weights, as illustrated in Figure 3.

**Figure 3:**
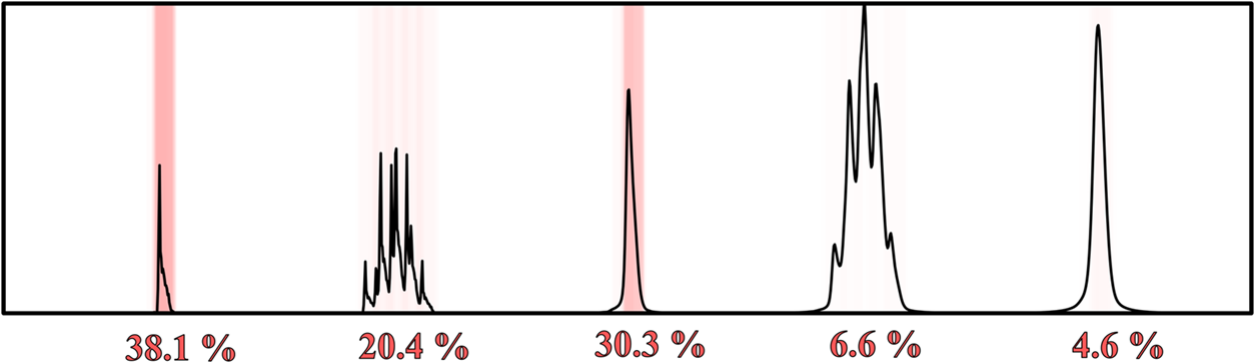
Localized attention paid to parts of the input spectrum - intensity is shown in red, with the per-multiplet summary weight written as a percentage below.

The RD and RH-Unet methods require manual selection of reference peaks used to extract information about distortion. To the contrary, our model retrieves this information from all the peaks and automatically weights them according to their usefulness. Consequently, the extracted information is more complete and precise. It is then aggregated and redistributed over the entire spectrum to reconstruct every peak, whether a singlet or a multiplet.

Our network also has an auxiliary decoder comprising two fully connected layers with a ReLU activation in between. It tries to reproduce a response function that distorts the spectrum, thus forcing the network to learn meaningful features. This decoder is used only in the training phase, as explained in Section 2.4.

### 2.4 Hybrid Training

The presented network is trained by passing distorted spectra as input and requesting that the outputs be close to their undistorted counterparts. However, collecting pairs of distorted and undistorted spectra experimentally and in sufficiently large amounts is virtually impossible. Therefore, we propose a hybrid training procedure that incorporates both experimental and synthetic elements. It is based on the assumption that the effect of magnetic-field inhomogeneity is described by a convolution of the ideal spectrum with a certain response function. The RD and RH-Unet methods effectively assume the same.

In a series of calibration measurements, we collect an extensive library of possible response functions for the given machine. We then synthetically generate ideal spectra containing random Lorentzian–Gaussian peaks, convolve them with randomly chosen response functions from the library, and add white Gaussian noise. Such distorted spectra are passed to the neural network as input, while their ideal counterparts serve as the target output.

The following sections give the necessary steps to train the network. They include performing a series of calibration measurements (Section 2.4.1), extracting the response functions (Section 2.4.2), adjusting the frequency resolution (Section 2.4.3), generating the training data (Section 2.4.4), and finally the actual training (Section 2.4.5). We describe the general procedure and also give some illustrative details of our exemplary implementation.

#### 2.4.1 Measurement of the Calibration Spectra

To train the model, one must first perform a series of calibration measurements on the same spectrometer that the model is trained for. They consist in collecting spectra of a calibration sample that can be chosen arbitrarily but should contain a well-resolved and relatively narrow singlet. We used the chloroform sample described in Section 2.1 and acquired spectra from both our spectrometers (700 and 600 MHz).

First, the spectrometer should be shimmed for the calibration sample using any effective method—manual shimming, gradient shimming, etc. We used gradient shimming and obtained *Z*1 = 8868 and *Z*2 = −297 (700 MHz machine) and *Z*1 = −5949 and *Z*2 = 2118 (600 MHz machine) with the entire scale ranging from −32,768 to +32767 (arbitrary units). Currents in other shim coils were also adjusted but were not varied in later steps. With the optimal shim settings, the first spectrum of the calibration sample should be acquired; we call it the best-shimmed one.

Afterward, the user should collect mis-shimmed spectra reflecting a possibly large class of distortions that may appear due to magnetic-field inhomogeneities in the concerned machine. The measurements could be disturbed in any valid way, such as by displacing the sample, changing its volume, concentration, etc. In the present study, we vary only the values of *Z*1 and *Z*2 because this can be done automatically using a simple script (we used our own macros presented previously^40^). As demonstrated in Section 4, such a limited class of distortions is nevertheless sufficient for ShimNet to correct reasonable spectra disturbed by factors other than *Z*1 and *Z*2.

On the 700 MHz machine, we collected 10,201 = 101 × 101 calibration spectra for *Z*1 = 8768, 8770, ···, 8968 and *Z*2 = −397, −395, ···, −197. On the 600 MHz machine, we acquired 17,161 = 131 × 131 spectra for *Z*1 = −6079, – 6077,..., −5819 and *Z*2 = 1988, 1990,..., 2248. The delay between consecutive acquisitions was 7 s (including saving the data files). In total, the calibration experiment took ca. 33 h on the 700 MHz machine and ca. 64 h on the 600 MHz machine. For illustration, Figure 4 shows the CHCl_3_ singlet in the best-shimmed spectrum and in three exemplary mis-shimmed spectra from the 700 MHz spectrometer.

**Figure 4:**
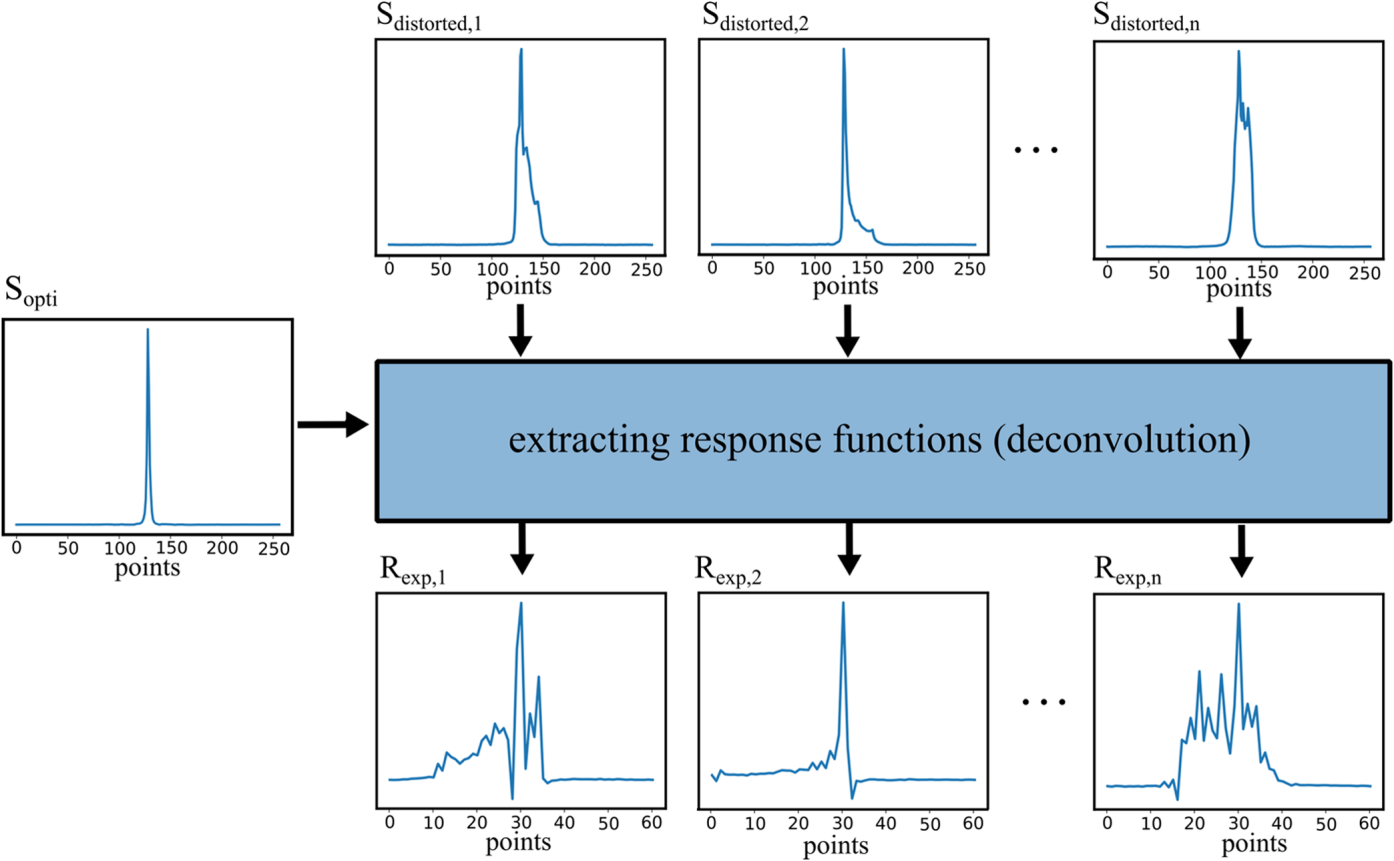
Extraction (deconvolution) of response functions from the distorted calibration spectra.

#### 2.4.2 Extraction of the Response Functions

The network is trained on hybrid spectra obtained by convolving synthetically generated Lorentzian–Gaussian peaks with response functions that describe the possible distortions due to magnetic-field inhomogeneities. In the present step, one response function is extracted from each calibration spectrum collected in Section 2.4.1.

A single response function is a vector of real values for *N* subsequent frequency points. *N* should be large enough to cover the broadest function obtained from the calibration experiment, as detailed in Section 2.4.3. Using the least-squares method, we explicitly fit the *N* values to reproduce a given calibration spectrum distorted due to magnetic-field inhomogeneities. Since the hybrid spectra are generated by convolving the response functions with perfect Lorentzian–Gaussian peaks, each function should be estimated by considering its convolution with such peaks. We checked that the CHCl_3_ singlet in our best-shimmed spectra is indeed very close to a perfect Lorentzian–Gaussian shape. Thus, we fit each response function by requesting that its convolution with the best-shimmed spectrum from the given machine reproduces the distorted spectrum under consideration.

Figure 4 shows response functions of *N* = 61 points extracted from our 700 MHz data. One can see that they assume zero values at both ends, which indicates that the adopted length is sufficient here. We also checked that the distorted calibration peaks were reproduced very well using the extracted functions. Finally, we verified that the functions changed rather smoothly with *Z*1 and *Z*2, indicating that the covered shim range contains no responses significantly different from the extracted ones so that the obtained set is, in a sense, complete.

However, note that the functions are asymmetrical and somewhat irregular, which confirms the need to determine them experimentally, as they do not follow theoretical approximations. In our settings, the procedure described in this section resulted in a library of 101 × 101 = 10,201 (700 MHz machine) and 131 × 131 = 17,161 (600 MHz machine) response functions that will be used in Section 2.4.4 to generate hybrid spectra for training our model.

#### 2.4.3 Adjustment of the Frequency Resolution

As detailed in Section 2.3, the ShimNet encoder and the entire autoencoder have receptive fields of 25 and 49 points, respectively. We roughly estimate that such a network can correctly capture the shape of a distorted peak if its width is below 100 points. On the other hand, for any information to be extracted from a peak, its full width at half maximum (FWHM) should not fall below 1 point. These observations impose some constraints that the NMR frequency resolution must fulfill to be compatible with our tool.

When adjusting the frequency step, Δ_*f*_ [Hz], the user should first estimate *W* [Hz], the maximum width of a response function expected to distort the measured spectra. It should be chosen so that the function fully fits into a frequency window of width *W* [Hz], as displayed in Figure 4. The value of *W* [Hz]­depends on the spectrometer and on the quality of hardware shimming. For our spectrometers, we estimate *W* to be ca. 21 Hz (700 MHz machine) and 23 Hz (600 MHz machine).

Afterward, the user should decide on FWHM_min_ [Hz] and FWHM_max_ [Hz], the minimum and maximum FWHM of the expected peaks without distortion. These values refer to singlets, whether standalone or members of a multiplet. For small molecules in liquid solutions, values of 0.4 and 4 Hz seem fairly universal.

The width of a distorted peak can be roughly estimated as the sum of its natural FWHM and *W*, the width of the response function. Therefore, the frequency step, Δ_*f*_ [Hz], should be adjusted to simultaneously fulfill the following two conditions

1 $${\mathrm{FWHM}}_{\min}>1\Delta_{f}$$

2 $$W+{\mathrm{FWHM}}_{\max}<100\Delta_{f}$$

They ensure that narrow undistorted singlets have FWHM above 1 point while broad and distorted ones have width below 100 points. We chose Δ_*f*_ ≈ 0.34 Hz (700 MHz machine) and Δ_*f*_ ≈ 0.29 Hz (600 MHz machine), which actually correspond to the default settings of our spectrometers for a ^1^H NMR experiment. The frequency step Δ_*f*_ [Hz]­pertains to the spectra directly fed to the network in Section 2.2 and used for extracting the response functions in Section 2.4.2.

Finally, the adopted Δ_*f*_ [Hz] also determines *N*, the length of the response function in points, via the simple dependence

3 $$W\leq N\Delta_{f}$$

Of course, *N* must be an integer and should preferably be chosen as odd due to some details of numerical convolution. We chose 61 and 81 points for the 700 and 600 MHz machines, respectively.

#### 2.4.4 Generation of the Training Data

A single hybrid spectrum for training the model is a convolution of a random response function from the library created in Section 2.4.2 with a synthetic spectrum obtained by summing Lorentzian–Gaussian peaks of the form

4 $$f\left(\omega \right)=\left(1-\gamma \right)H\frac{w^{2}}{w^{2}+{\left(\omega -\omega_{0}\right)}^{2}}+\gamma H\exp\left(-\frac{{\left(\omega -\omega_{0}\right)}^{2}}{2\sigma^{2}}\right)$$

Here, *H* is the total peak height while γ is the fraction that the Gaussian component has therein. Also, ω_0_ is the central peak frequency, *w* is the half-width at half-maximum for the Lorentzian part, and σ is the dispersion of the Gaussian part. We find it sufficient to assume equal widths for both components, which amounts to setting $w=\sigma \sqrt{2\log2}$ .

A synthetic spectrum contains a random number of peaks of the form (4) with randomly chosen parameters. These peaks could be independent singlets, but ShimNet trained on such simple spectra often fails to correctly shim multiplets. Thus, the training spectra should contain multiplets as well. Following the approximation of weak scalar coupling, we assume a multiplet to be a sum of equidistant and equally wide components of the form (4) with relative heights given by the Newton binomial coefficients, *h*_b_.

Figure 5 shows the training data generation scheme. To generate multiplets in an acceptably realistic way, we drew 10,000 molecules from the Enamine REAL Database^41^ complying with the Ro5 ^43^ and Veber^44^ criteria. For each molecule, we estimated all its NMR peak multiplicities, *m*, and relative intensities, *h*_*i*_, using the Ambit-HNMR software by Kochev et al.^42^ In this approach, singlets are treated as one-fold multiplets while the relative intensity of each multiplet equals the number of resonating protons. The described procedure resulted in a library of 8720 multiplets.

**Figure 5:**
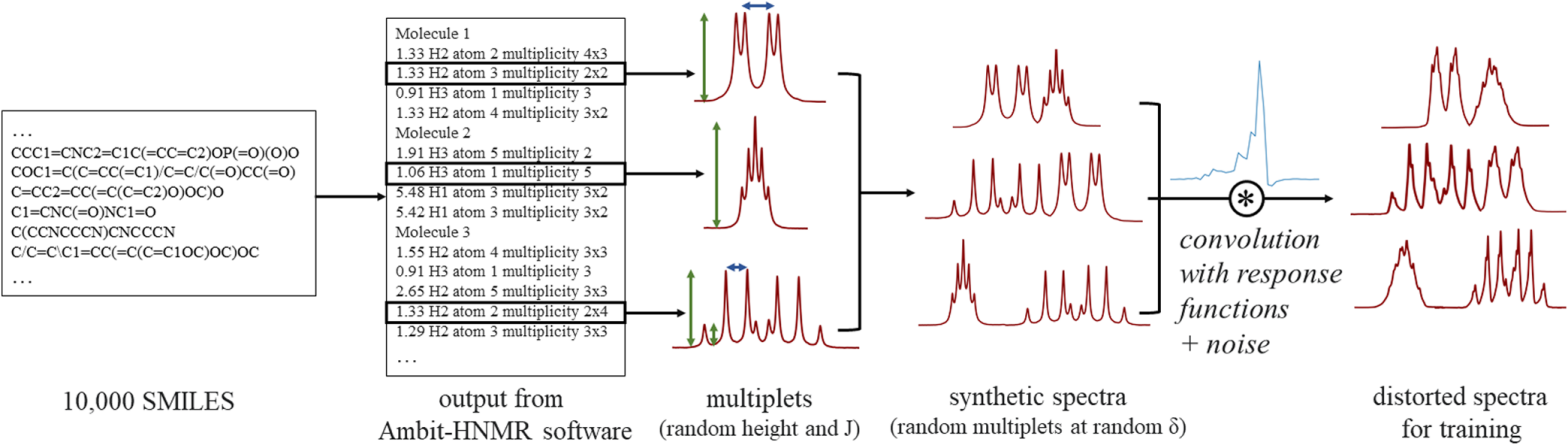
Training data generation scheme. SMILES codes of 10,000 molecules from the Enamine REAL Database^41^ are used to generate NMR multiplets using the Ambit-HNMR software.^42^ Then, randomly scaled random multiplets are placed at random chemical shifts, forming “clean” synthetic spectra. Finally, the synthetic spectra are convolved with the shim coil response functions to form the distorted spectra for training.

To generate a synthetic spectrum, *M* multiplets are randomly selected from the library and placed at random frequencies, ω_0_. The spacing between multiplet components is defined by randomly selecting the *j*_1_ and *j*_2_ coupling constants, for each multiplet separately. The width, *w*, of each component in a multiplet is the product of a random width, *w_m_*, drawn separately for each multiplet and a random factor, *w_s_*, chosen for the entire spectrum. The height, *h*, of each component within a multiplet is the product of the binomial coefficient, *h_b_*, the relative intensity, *h_i_*, a random factor, *h_m_*, chosen separately for each multiplet, and another factor, *h_s_*, drawn randomly for the entire spectrum. Finally, the Gaussian fraction, γ, is randomly assigned to each multiplet separately. This is summarized in Table 1, which also gives the range of each parameter.

**Table 1:** Parameters Characterizing a Single Hybrid Spectrum Generated for Training Our Model^a^

	symbol	min	max	scope	source
number of peaks	*M*	2.00	5.00	spectrum	random
central frequency (Hz)	ω_0_	–279.02	279.02	multiplet	random
multiplicity	*m*	1	7	multiplet	Ambit-HNMR
coupling constants (Hz)	*j*_1_, *j*_2_	0.00	15.00	multiplet	random
component width (Hz)	*w_m_*	1.00	2.00	multiplet	random
width factor	*w_s_*	0.20	1.00	spectrum	random
binomial coefficient	*h_b_*	0.0039	1.00	component	multiplicity
relative intensity	*h_i_*	1.00	3.00	multiplet	Ambit-HNMR
height factor	*h_m_*	0.50	4.00	multiplet	random
height factor	*h_s_*	0.50	2.00	spectrum	random
Gaussian fraction	γ	0.00	1.00	multiplet	random
response function indices	*Z*_1_, *Z*_2_			spectrum	random
noise amplitude	N	0.00	0.02	spectrum	random

a Parameters with component scope are different for each component of a multiplet. Parameters with Multiplet scope are common for each component of a multiplet but different for each multiplet in a spectrum. Parameters with Spectrum scope pertain to the whole spectrum.

The adopted training parameters are appropriate for high-field NMR spectra of liquid solutions of small molecules at concentrations of 0.5 mM and above. This corresponds to typical samples routinely measured by chemists. Of course, the network can be retrained for different settings if, e.g., the users are interested in spectra of middle-sized molecules, mixtures, or solid-state samples. Notably, we performed preliminary tests of the current network in application to spectra deviating from the mentioned parameters, and the results were still satisfactory, i.e., some of the distortions were corrected and no significant artifacts were introduced. Yet, one should be careful with too much “extrapolation” from the training data.

More realistic multiplets could be generated by assuming strong couplings and using more advanced software, such as SPINACH.^45^ However, such procedures would be prohibitively slow for our purposes because we generate synthetic spectra in real time during the training process, as explained in Section 2.4.5. We find the current multiplets a plausible compromise between more complex ones and plain singlets. Note that the synthetic peaks are placed at random frequencies so that different structures may overlap. This partially prepares the neural network for more complex cases that can appear in experiment.

All in all, the half-width at half-maximum, *w*, for each component of a multiplet varies from 0.2 to 2 Hz, which corresponds to the FWHM range discussed in Section 2.4.3 and fairly universal for small molecules in liquid solutions. The peak height *h* most often falls within the range from 0.022 to 3.34 which follows from the minimum and maximum values of *h_b_*, *h_i_*, *h_m_*, and *h_s_*. Higher peaks occasionally arise if several structures overlap. The neural network trained on such a height range is prepared to process only peaks within that range. As mentioned in Section 2.2, our program automatically rescales any spectrum provided in the prediction phase to keep the highest peak within the proper range of peak height.

Once a synthetic spectrum is generated, it is convolved with a randomly chosen response function from the library created in Section 2.4.2 to simulate the distortion caused by magnetic-field inhomogeneity. Finally, white Gaussian noise of random dispersion N is added to form the hybrid spectrum used for training; refer to Table 1 for the adopted range of N . Figure 5 gives some examples of synthetic (ideal) spectra and their hybrid (distorted and noisy) counterparts. In the training process, a hybrid spectrum is passed to the neural network as input, while its underlying synthetic spectrum serves as the reconstruction target. In fact, also the corresponding response function is used for training, as described in Section 2.4.5.

#### 2.4.5 Training the Network

For training the ShimNet, we generate spectra of 2048 points, significantly shorter than in typical NMR measurements. It is possible to train the model on such spectra and then use it for arbitrary other lengths because the network is fully convolutional and processes inputs of any size using the same parameter values. On the other hand, shorter inputs allow for larger batches, which expose the network to a greater diversity of response functions. This reduces undesired gradient fluctuations and stabilizes the training process.

We train the network by minimizing a loss that measures the deviation of the actual model output from the desired one for batches of training data. Let *S*_trg_ and *S*_inp_ be a batch of synthetic (ideal) spectra and a batch of the corresponding hybrid (distorted and noisy) ones, both generated according to Section 2.4.4. The hybrid spectra are passed to the network, which returns a batch of reconstructed ones, denoted here as *S*_out_. As mentioned in Section 2.3, our network has an auxiliary decoder that attempts to reconstruct the response functions from the hybrid spectra. Let us denote its output as *R*_out_ and let *R*_trg_ be a batch of actual response functions used to transform *S*_trg_ into *S*_inp_.

We minimize the value of a loss function defined as

5 $$L=\frac{1}{K}\left(\left|S_{\mathrm{trg}}-S_{\mathrm{out}}{|}^{2}+\right|R_{\mathrm{trg}}-R_{\mathrm{out}}{|}^{2}+|S_{\mathrm{inp}}-S_{\mathrm{out}}\times R_{\mathrm{out}}{|}^{2}\right)$$

where *K* is the number of spectra in a batch and * denotes convolution. The first part, dubbed reconstruction loss, makes the reconstructed spectra close to the synthetic ones, which corresponds to the main task of ShimNet. The second part, dubbed distortion loss, requests that the auxiliary decoder correctly reconstructs the response function from the global features produced by the attention layer. This indirectly ensures that the attention and encoder properly extract the information about the global distortion from the input spectrum. The last part of the loss function requests that the reconstructed spectrum distorted by the reconstructed response function yields the input hybrid spectrum.

The third term uses the noisy hybrid spectrum as a target, while the first one uses the synthetic spectrum without noise. We argue that this slight contradiction has some regularization effect that may deteriorate the performance on generated data but better prepares the model for the forcibly less ideal spectra from the experiment. Indeed, we empirically found that including the second and third terms of the loss function does slightly but visibly improve the reconstruction quality for experimental data.

We do not maintain a finite set of training examples but generate the synthetic and hybrid spectra independently for each batch during the training process. This approach, known as online learning, offers virtually infinite amounts of training data, which greatly reduces overfitting. We do not use other regularization techniques, such as dropout or weight decay.

We minimize the loss using the Adam optimizer,^46^ one of the most popular extensions to the gradient descent algorithm. Throughout the training process, we gradually increase the batch size, simultaneously reducing the learning rate, as specified in Table 2. Initially, this results in stronger gradient fluctuations and allows the algorithm to escape from local minima etc. In later phases, it reduces the fluctuations and lets the algorithm converge more steadily. As indicated in Table 2, the network has seen a total of 40 M generated spectra in the training process.

**Table 2:** Batch Sizes and Learning Rates Used for Training the ShimNet

number of spectra	batch size	learning rate
1.6 M	64	0.0010
25.6 M	512	0.0010
12.8 M	512	0.0005

We train ShimNet on a single PC with 20 CPU cores and a GeForce RTX 3060 GPU with 12 GB of memory. The training takes ca. 20 h, yet reasonable performance is achieved much quicker (<1/10 of the full training time), which can be beneficial for users with limited computational resources.

Figure 6 shows the reconstruction loss in the function of the batch number. Figure 7 shows three sample reconstructions of spectra and of their corresponding response functions.

**Figure 6:**
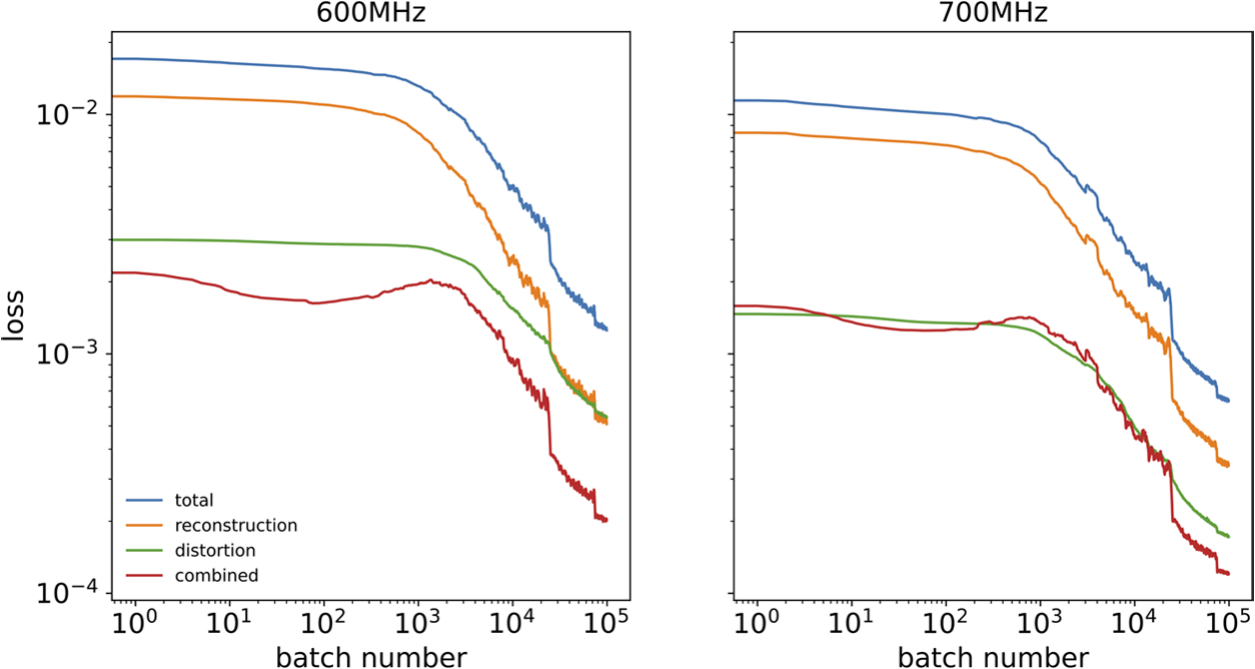
Training loss and its components smoothed over a 1000-batch window.

**Figure 7:**
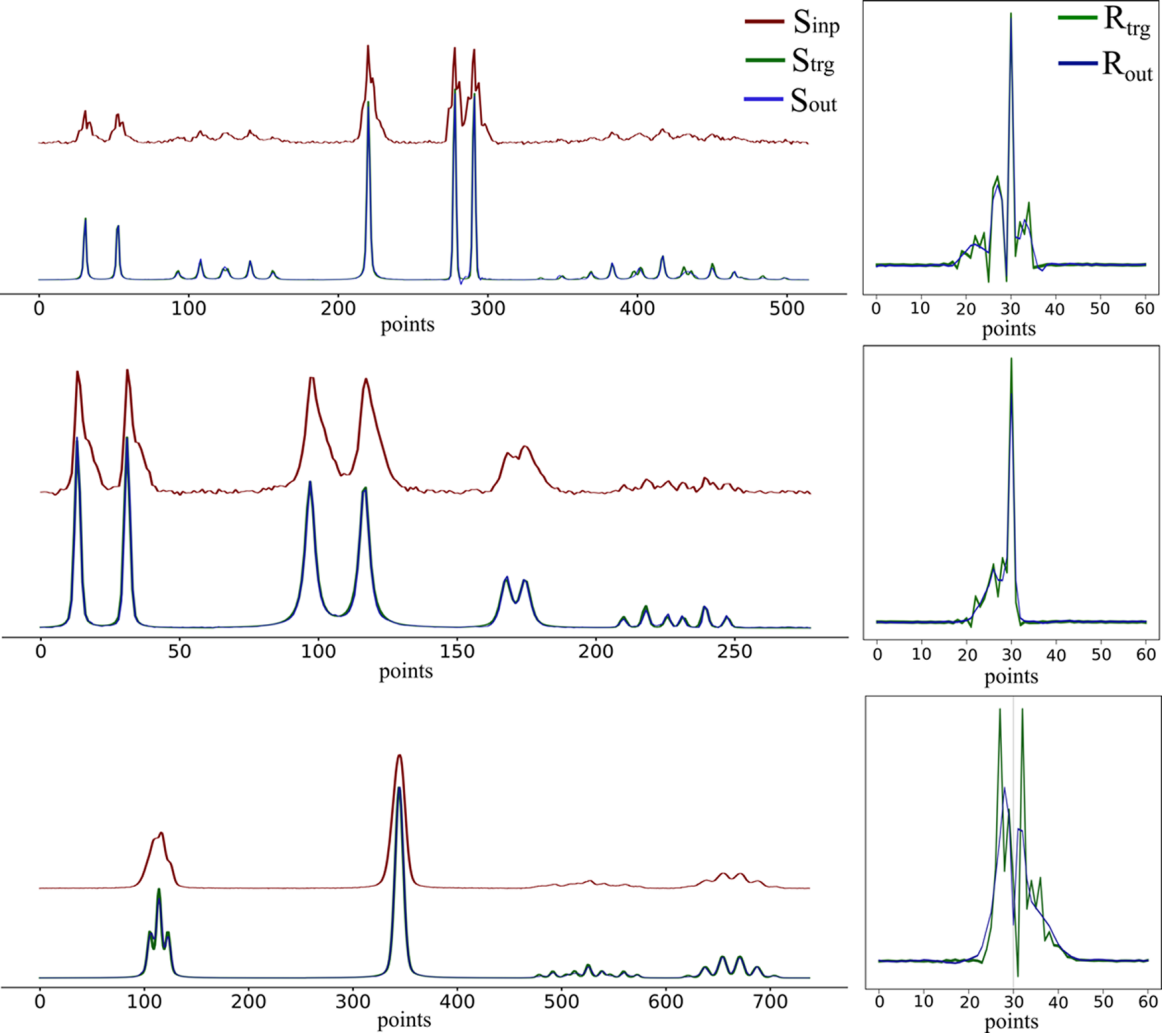
Reconstruction of three sample spectra (left) and of the corresponding response functions (right). *S*_inp_ is shown in red, *S*_out_ and *R*_out_ are shown in blue, while *S*_trg_ and *R*_trg_ are shown in green.

The presented results show the excellent performance of ShimNet on the training data. This is the necessary condition that each machine-learning model must fulfill. In online learning, each batch of synthetic spectra is different from all the previous ones. Thus, the presented results also indicate good performance for all other spectra generated with the same set of response functions. A full assessment for new test spectra would require an independent set, acquired for the same machine but in a different way. However, evaluating the model on artificially generated data is only indicatory, so we skip this step and proceed to experimental spectra, which are of the real interest here.

## 3 ShimNet Software Tool

Section 2 describes the neural network at the core of ShimNet. To facilitate its usage, we provide a complete software package that wraps the network in convenient Python scripts. It is freely available from github at https://github.com/center4ml/shimnet and as the web service at https://huggingface.co/spaces/NMR-CeNT-UW/ShimNet. There is a script for extracting the response functions, for training the network, and for performing the actual shim correction. They are thoroughly described in the readme file. However, here we give some basic overview of the latter script to give the reader an idea of using ShimNet in practice.

Before collecting the calibration spectra, the user should follow Section 2.4.3 to choose an appropriate frequency step, Δ_*f*_[Hz], and save it in a configuration file. This file is read by all the scripts in the package. In particular, they train the network for this particular Δ_*f*_[Hz], as detailed in Section 2.4.3. If technical staff trains the model in a laboratory, they should provide the configuration file to the end user.

Every preprocessed spectrum should be saved in a text format specified in the readme file. The format is simple and natively handled by popular NMR software like Mnova (Mestrelab Research, S. L., Spain) and TopSpin (Bruker, USA). It carries information about the spectrum frequency step, which can be arbitrary. The correction script automatically resamples the input spectrum to the resolution of Δ_*f*_[Hz], required by the neural network.

The script also normalizes the spectrum values to fit into the range of peak heights for which the network was trained, as detailed in Section 2.4.4. Specifically, the highest value is scaled to 16 and all the other are transformed proportionally. The value of 16 complies with the training parameters in Table 1 and was adjusted empirically to achieve the highest reconstruction quality.

The spectrum prepared in this way is passed to the neural network, which performs the actual shim correction. The result is scaled back to regain the original height of the tallest peak and resampled to the original frequency resolution. Thus, the user does not need to care about normalization or frequency resolution of the spectra passed to the ShimNet correction script. In particular, zero-filling can be used freely as part of the preprocessing routine.

## 4 Results and Discussion

To test our solution on experimental data, we acquire the best-shimmed and distorted spectra of several samples, as described in Section 2.1. Then, we correct the distorted spectra using ShimNet, and compare the results against the best-shimmed spectra. We consider distortions due to magnetic-field inhomogeneity caused by various factors, as specified below.

To correct spectra, we use the set of ShimNet parameters obtained in Section 2 as a result of training the model on response functions for the same machine. Since the training conditions are adjusted for small molecular compounds in deuterated solvents, we test ShimNet for this kind of cases. Specifically, we consider seven samples and various sources of magnetic-field inhomogeneities as listed in Table 3. Apart from being measured on 700 and 600 MHz machines, the samples differ in their compositions, concentrations, and volumes, reflecting the diversity of possible experimental conditions in several respects. For each sample separately, we first placed it properly in the spinner, applied the full gradient-shimming procedure, and collected the best-shimmed spectrum. Then, we induced inhomogeneities listed in the Table 3 and acquired the distorted spectrum.

**Table 3:** Samples and the Corresponding Spectral Distortions or Sources of Magnetic-Field Inhomogeneity Used to Test ShimNet on Experimental Data

sample	inhomogeneity/distortion
14 mM azarone in MeOD, 650 μL, 600 MHz	Z1, Z2 shim changed
14 mM azarone in MeOD, 650 μL, 600 MHz	Z1, Z2, Z3, Z4 shim changed
14 mM azarone in MeOD, 650 μL, 600 MHz	X shim changed
20 mM azarone in MeOD, 670 μL, 700 MHz	20 μL volume decrease
3 M styrene in CDCl_3_, 700 μL, 700 MHz	0.5 mm vertical sample shift
2.24 mM sodium butyrate in D_2_O, 650 μL, 700 MHz	Shims settings from another sample
0.78 mM Cresol Red in DMSO, 600 μL, 600 MHz	Shims settings from another sample
5.40 mM geraniol in acetone-d, 600 μL, 600 MHz	1 mm vertical sample shift
3 M 2-ethylnaphthalene in CDCl_3_, 700 μL, 700 MHz	1 mm vertical sample shift

The following sections detail the tests listed in Table 3. The first three consist in manual misadjustment of electric currents in the shim coils (Section 4.1). The next four involve distortions caused by various factors likely to appear in a typical experiment (Section 4.2). The last two tests pertain to large distortions that are actually beyond the range that the shimator was trained for (Section 4.3).

### 4.1 Artificially Misadjusted Shims

As explained in Section 2, we train our model on response functions corresponding to misadjusted currents in the *Z*1 and *Z*2 shim coils. It is therefore natural to first test its performance on experimental spectra distorted by manual misadjustment of these two currents. It is also interesting to see how the model handles magnetic-field inhomogeneities induced by other shim coils.

To address these points, we carried out three tests for 14 mM azarone solution in MeOD, as listed in the top three rows of Table 3. We first changed only *Z*1 and *Z*2 respectively by +30 and +30, then *Z*1, *Z*2, *Z*3, and *Z*4 respectively by −20, −40, −200, and −200, and finally only *X* by −50. Each set of changes was applied separately with respect to the best-shimmed case. We then reconstructed the distorted spectra using ShimNet and, for comparison, also using the RH-Unet and RD methods cited in Section 1. We used an RD tool implemented in MNova 15.0 and the original RH-Unet software provided by the Authors. Figure 8 shows all the corresponding distorted, corrected, and best-shimmed spectra.

**Figure 8:**
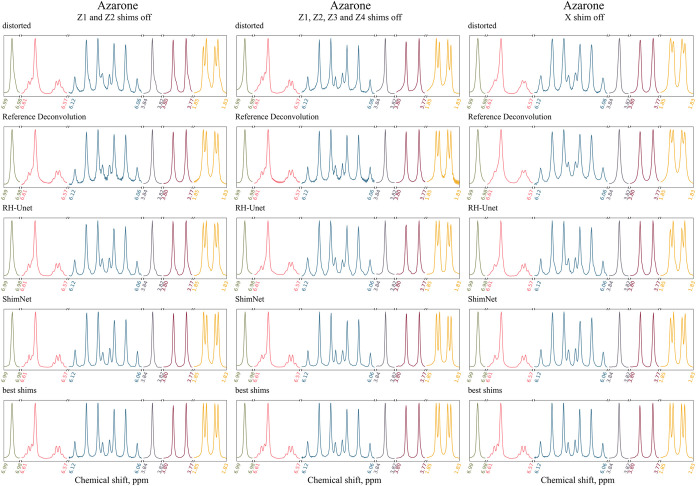
Spectra of 14 mM azarone solution in MeOD acquired on an Agilent 600 MHz spectrometer and corrected by RD, RH-Unet, and ShimNet. The spectrum was distorted by Δ*Z*1 = +30, Δ*Z*2 = +30 (left), Δ*Z*1 = – 20, Δ*Z*2 = – 40, Δ*Z*3 = – 200, Δ*Z*4 = – 200 (middle), and Δ*X* = – 50 (right). The best-shimmed spectrum acquired before applying the distortion is displayed in each column for comparison. Six peak groups are shown in different colors and normalized to the same height.

Our exemplary misadjustment of *Z*1 and *Z*2 alone manifests itself in a slight peak broadening accompanied by pronounced tails on the right peak slopes. These artifacts are fully removed by each method. However, the details of the doublets between 1.85 and 1.83 ppm and of the quartets between 6.61 and 6.57 ppm are best reconstructed by ShimNet. Additionally, RD produces some artificial noise in the baseline. The same conclusions hold for the sample perturbation of *Z*1, *Z*2, *Z*3, and *Z*4, which yields different kinds of tails on the left peak slopes. Finally, the deviation in *X* shim makes the distorted peaks look more Gaussian and broader than they actually are in the best-shimmed spectrum. These artifacts are best corrected by ShimNet, which also yields the most reliable reconstruction of the aforementioned multiplets. As to the latter point, however, even the ShimNet results are less accurate compared to the cases distorted by the *Z* shims alone.

We also performed other tests based on manual changes of electric currents in the shim coils and got similar results. In conclusion, the ShimNet correction always significantly improves the distorted spectrum. Oftentimes, the reconstruction is virtually perfect and even if not, ShimNet usually outperforms other existing methods by a larger or smaller margin. The least accurate results are obtained when the magnetic field is disturbed by currents in the *X* or *Y* coils, apparently because the model is trained by misadjusting only the *Z*1 and *Z*2 shims. In principle, one could extend the calibration loop of Section 2 by including more shims at the expense of longer acquisition times or sparser sampling of the response functions.

### 4.2 Typical Experimental Distortions

In experimental practice, the NMR spectroscopy assumes that any inhomogeneity of the magnetic field can be corrected by adjusting currents in the shim coils. Consequently, any inhomogeneity could be simulated by misadjusting those currents so that varying the currents theoretically covers all the possible cases. However, it is instructive to explicitly test the model for distortions that may actually arise in a real experiment. To do so, we performed four measurements corresponding to the middle rows in Table 3. They involve slight sample displacement, inaccurate volume adjustment, or using shim-coil settings from a different sample.

First, we acquired a spectrum of 20 mM azarone solution in MeOD distorted by a 20 μL reduction in the sample volume and a spectrum of 3 M styrene solution in CDCl_3_ distorted by a 0.5 mm upward shift in the sample position (measured on the standard position gauge). Figure 9 shows the distorted, reconstructed, and best-shimmed spectra for these two cases. For azarone, the peak distortion is quite symmetrical, while for styrene, it is visibly asymmetrical with pronounced tails to the left. In both cases, the ShimNet correction is very good, leaving only tiny baseline deformations close to the peaks.

**Figure 9:**
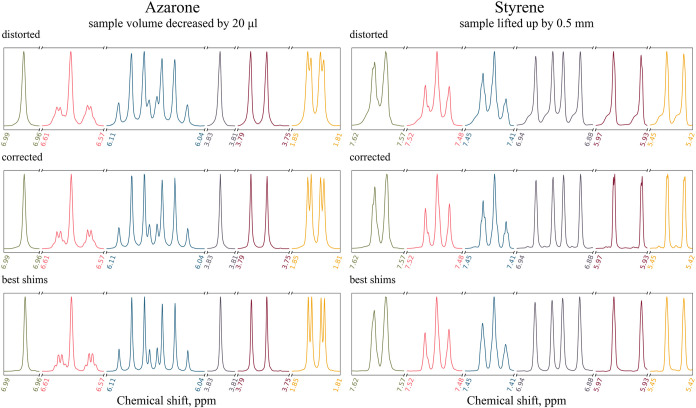
Spectra of 20 mM azarone solution in MeOD (left) and of 3 M styrene solution in CDCl_3_ (right) acquired on an Agilent 700 MHz spectrometer and corrected by ShimNet. The azarone and styrene spectrum was distorted by 20 μL decrease in the sample volume and by 0.5 mm upward shift of the sample in the spinner, respectively. The best-shimmed spectrum acquired before applying the distortion is displayed in each column for comparison. Six peak groups are shown in different colors and normalized to the same height.

Interestingly, the reconstructed styrene spectrum reveals subtle multiplet patterns which indeed follow from the molecule structure but remain hidden even with optimal hardware shimming. The effect is best visible for the peaks between 7.45 and 7.41 ppm. However, this is not only thanks to the ShimNet correction itself but also because the patterns are better pronounced in the distorted spectrum than in the best-shimmed one.

In massive measurements of many similar samples, it is common to apply an automatic gradient shimming using one general shim map instead of preparing a dedicated map for each sample. Even with this simplification, the shimming procedure is lengthy compared to the actual acquisition time of an FID. In a typical setup, gradient shimming takes tens of seconds, while a single-scan NMR experiment takes a second or two. Thus, acquiring massive, serial data sets without reshimming between samples would be tempting. We decided to test ShimNet in such a scenario.

We first shimmed 700 MHz spectrometer for a sample of 700 μL glucose in D_2_O, then collected a spectrum of 2.24 mM sodium butyrate solution in D_2_O. We repeated a similar experiment on 600 MHz spectrometer collecting a spectrum of 600 μL 0.78 mM Cresol Red solution in DMSO using shim set from 600 μL styrene in CDCl_3_. Importantly for the considerations below (Section 4.2), the naturally hygroscopic DMSO used to prepare Cresol Red sample contained some H_2_O giving a large peak. Right columns of Figures 10 and 11 show the distorted, corrected, and best-shimmed spectra of sodium butyrate and Cresol Red, respectively.

**Figure 10:**
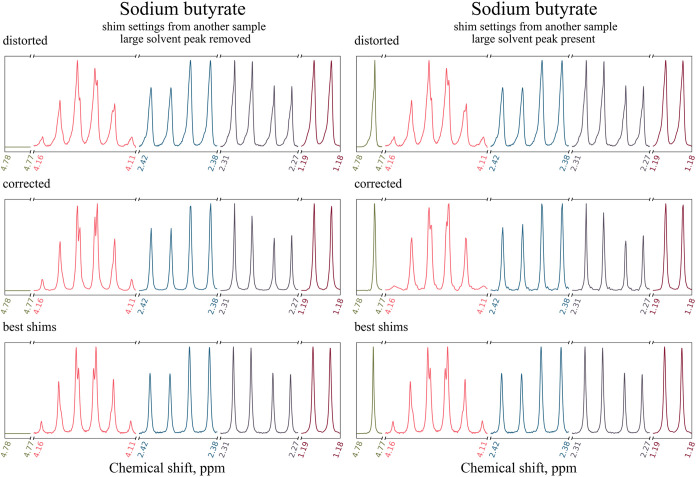
Spectra of 2.24 mM sodium butyrate solution in D_2_O acquired on a 700 MHz spectrometer an corrected by ShimNet. The spectrum was reconstructed in the presence of the strong H_2_O peak (left column) and with that peak removed (right column). The best-shimmed spectrum acquired before applying the distortion is displayed in each column for comparison. Five peak groups, including the H_2_O peak between 4.78 and 4.77 ppm, are shown in different colors and normalized to the same height.

**Figure 11:**
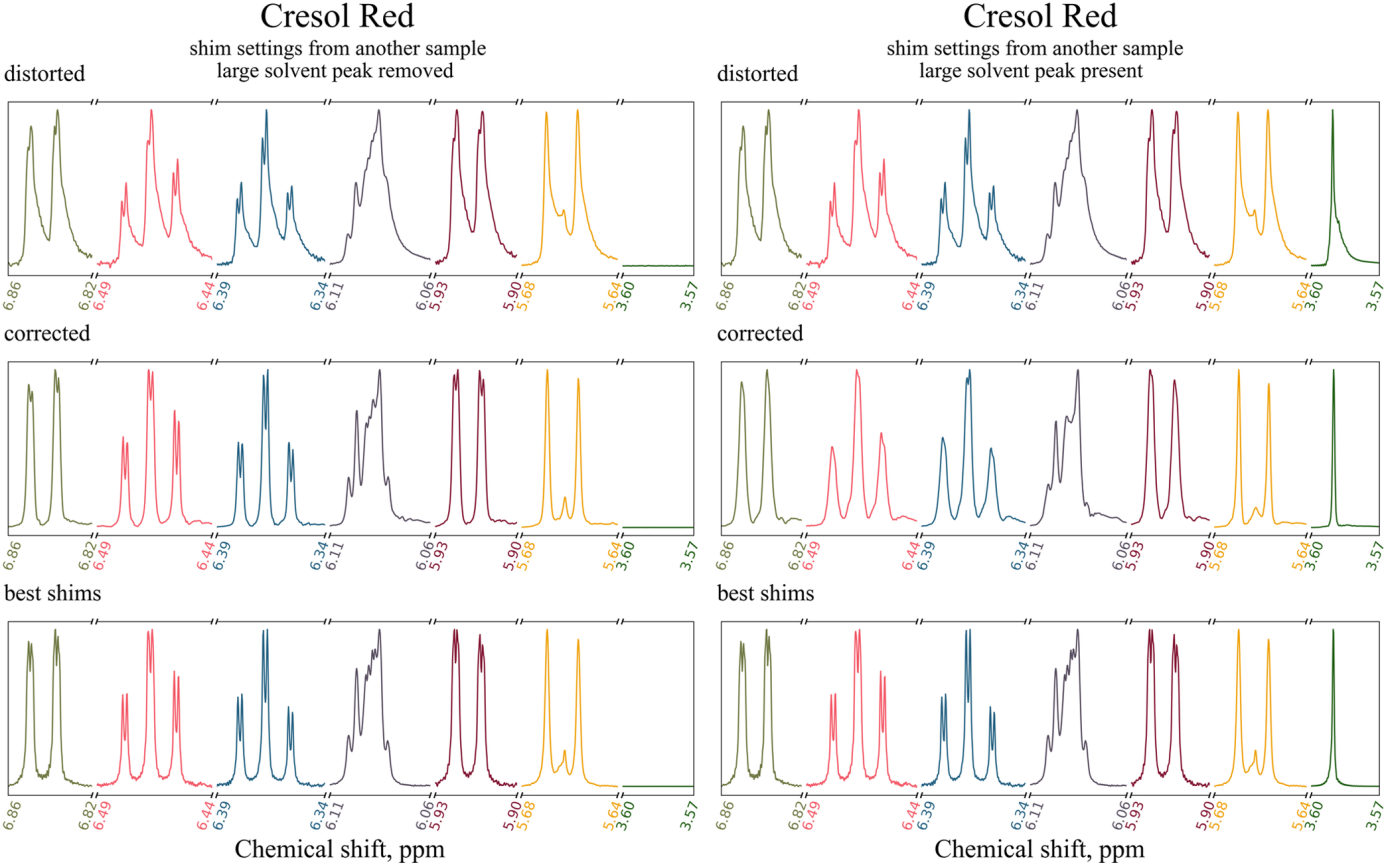
Spectra of 0.78 mM Cresol Red solution in DMSO containing some H_2_O contamination, acquired on a 600 MHz spectrometer an corrected by ShimNet. The spectrum was reconstructed in the presence of the strong H_2_O peak (left) and with that peak removed (right). The best-shimmed spectrum acquired before applying the distortion is displayed in each column for comparison. Five peak groups, including the H_2_O peak between 3.60 and 3.57 ppm, are shown in different colors and normalized to the same height.

The distorted peaks of sodium butyrate exhibit only slight broadening and tails to the left, while the distorted peaks of Cresol Red are significantly broadened with pronounced tails to the right and include complex structures like the compound multiplet between 6.11 and 6.06 ppm. In both cases, the ShimNet correction removes the broadening and asymmetric tails. Remarkably, even the complex peak in Cresol Red is properly reconstructed with only minor glitches.

As discussed in Sec. 2, ShimNet is trained on synthetic spectra that cover a dynamic peak-height range of 1:220 (90% of data), which is usually appropriate for small molecular compounds dissolved in deuterated solvents. Therefore, the reconstruction quality may deteriorate if the input spectrum contains excessively high peaks, often resulting from residual amounts of nondeuterated solvents like H_2_O. We investigated the influence of such contamination on the spectra of sodium butyrate and Cresol Red.

The results discussed above and presented in the right columns of Figures 10 and 11 were obtained by removing the H_2_O peaks located between 4.78 and 4.77 ppm for sodium butyrate and between 3.60 and 3.57 ppm for Cresol Red. With those peaks removed, the spectra exhibit peak-height ranges of, respectively, 1:21 and 1:12.5, which fit within the training range of ShimNet.

In the presence of the H_2_O peaks, the height ratios attain 1:123.5 for sodium butyrate and 1:180 for Cresol Red. Such high ratios are represented only in 16.3% and 12.1% of spectra, respectively. We nevertheless attempted a reconstruction without removing the H_2_O peak, and the results are displayed in the left columns of Figures 10 and 11. As expected, the overall reconstruction quality is visibly worse, particularly for the more difficult case of Cresol Red. Therefore, we recommend removing large solvent peaks before ShimNet correction.

### 4.3 Large Distortions

Every postacquisition shimming method has its limitations. In the case of ShimNet, they come from the neural-network architecture, from the set of response functions used for calibration, and from other training parameters. As a consequence, very large distortions will not be corrected fully. Nevertheless, we checked how ShimNet behaves under such circumstances by performing two tests from the bottom rows of Table 3.

We acquired a spectrum of 3 M 2-ethylnaphthalene solution in CDCl_3_ on the 700 MHz machine and a spectrum of 5.40 mM geraniol solution in acetone-d on the 600 MHz machine, both distorted by a 1 mm upward shift of the sample in the spinner. Figure 12 shows the distorted, corrected, and best-shimmed spectra for 2-ethylnaphthalene and geraniol. The subtle multiplet structure is fully lost in the distorted spectra, which is best visible between 1.64 and 1.60 ppm for 2-ethylnaphthalene and between 5.16 and 5.09 ppm for geraniol.

**Figure 12:**
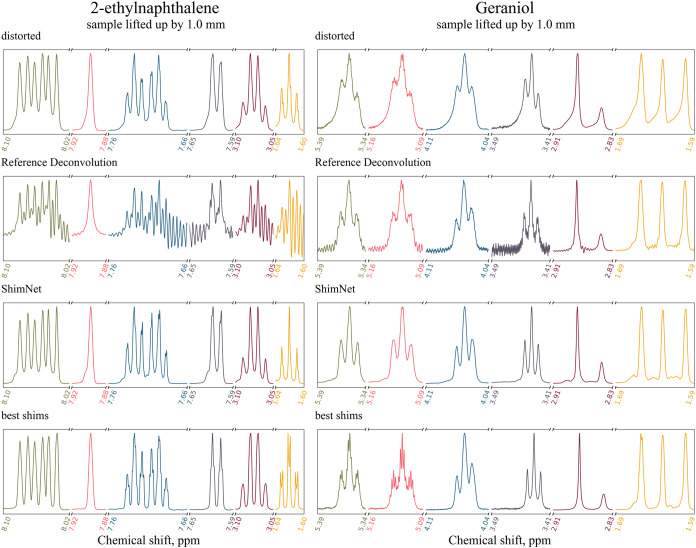
Spectra of 3 M 2-ethylnaphthalene solution in CDCl_3_ acquired on a 700 MHz spectrometer (left) and of 5.40 mM geraniol solution in acetone-d acquired on a 600 MHz spectrometer (right), both corrected by ShimNet and the RD. Both spectra were distorted by 1 mm upward shift of the sample in the spinner. The best-shimmed spectrum acquired before applying the distortion is displayed in each column for comparison. Six peak groups are shown in different colors and normalized to the same height. For RD processing, the peaks at ca. 7.90 ppm (2-ethylnaphthalene) and ca. 2.88 ppm (geraniol) have been selected as references.

The ShimNet correction cannot recover the details of these multiplets and the overall reconstruction quality is visibly worse than in the previous tests. However, ShimNet still improves the spectra with respect to the distorted originals and does not introduce any artifacts that could be mistaken for nonexistent structures. Thus, even for excessively large distortions, the result is still within reasonable limits.

Notably, the Reference Deconvolution method fails to correct both spectra, introducing oscillatory baseline distortions. The probable source of error is the incorrect automatic guess of reference peak parameters, a common problem when applying an RD method (we used an RD tool implemented in MNova 15.0, with default settings).

As a rule of thumb, the distortion is too strong for the effective use of ShimNet if the multiplet maxima (peaks) are smoothed out to such an extent that they are indistinguishable. In other words, the response function cannot be too broad compared to the distances between peaks in a spectrum. Our method shares this limitation with RH-Unet, which requires peak-picking before the correction. Moreover, the effectiveness of the ShimNet is significantly worse, if the width of the response function exceeds the width of the broadest response functions for which ShimNet was trained. This is the case of geraniol spectra from Figure 12.

## 5 Conclusions

ShimNet is a postacquisition shimming tool for removing spectral distortions caused by magnetic-field inhomogeneities in an NMR spectrometer. It is based on a neural network incorporating the attention mechanism into the autoencoder architecture. The model learns from a set of response functions, which must be determined for a given spectrometer in a series of measurements. The calibration experiment and the training process may last up to days, but once finished, the model reconstructs every spectrum in a split second. Contrary to other methods, the correction requires no user involvement apart from providing the input. Thus, the method is particularly well suited for massive measurements.

We checked in a series of experimental tests that ShimNet usually outperforms other methods, whether standard or based on machine learning. Oftentimes, the correction is virtually perfect and even if not, the model always improves the distorted spectrum quite significantly.

We believe that the presented version is reliable enough to be used in industry and academia. However, several improvements can be envisaged. The current procedure of extracting the response function assumes that an optimally shimmed spectrometer provides ideal Lorentzian–Gaussian peaks. Although this is true up to a very good approximation in our case, one can release this constraint by explicitly fitting a Lorentzian–Gaussian curve to peaks of the calibration sample. One could also enlarge the collected set of response functions by randomly disrupting them with Gaussian processes as a form of data augmentation. The synthetic spectra are now perfectly phased, but to better resemble experimental data, artificial phase perturbations could be introduced. Finally, the model could be extended to allow nonexponential apodization.

In principle, ShimNet should be trained for each spectrometer separately, but we observed that cross-machine applications also yield plausible results. This brings an idea that a single model could be trained for a whole series of spectrometers already by the manufacturer and even shipped with the machines. This should be possible at least in the case of spectrometers dedicated for metabolomics or food profiling because they are very well standardized. Such a solution would exempt the customers from performing the calibration measurements and from training the model themselves.

## Data Availability

The experimental data is available from https://github.com/center4ml/shimnet (sample_data folder). The open-source Python code is freely available from https://github.com/center4ml/shimnet. The web service is available at https://huggingface.co/spaces/NMR-CeNT-UW/ShimNet.
