# Correction to “ShimNet: A Neural Network for Postacquisition Improvement of NMR Spectra Distorted by Magnetic-Field Inhomogeneity”

**DOI:** 10.1021/acs.jpcb.5c04434

**Published:** 2025-07-11

**Authors:** Sylwia Jopa, Marek Bukowicki, Alexandra Shchukina, Krzysztof Kazimierczuk

The authors change the sentence in the Acknowledgments section to clarify Dr. Przemysław Olbratowski’s contribution to the paper. The complete revised Acknowledgments section reads as follows:

